# Evaluation of In Vitro Effects of Fosfomycin in Combination With Amikacin and Ciprofloxacin Against Multidrug-Resistant Escherichia coli Isolates

**DOI:** 10.7759/cureus.104921

**Published:** 2026-03-09

**Authors:** Swadhin Choudhury, Kumudini Panigrahi, Subhra Snigdha Panda, Dipti Pattnaik, Jyoti Prakash Sahoo

**Affiliations:** 1 Microbiology, Kalinga Institute of Medical Sciences, Bhubaneswar, IND; 2 Pharmacology, Kalinga Institute of Medical Sciences, Bhubaneswar, IND

**Keywords:** amikacin, biofilm layer, ciprofloxacin, drug-interaction, drug synergism, escherichia coli infection, extended-spectrum beta-lactamase (esbl), fosfomycin sensitivity, in vitro study, multidrug resistance (mdr)

## Abstract

Background and objectives: Multidrug-resistant (MDR) *Escherichia coli *is a global concern. *E*.* coli *exhibits resistance through extended-spectrum beta-lactamase (ESBL) production, efflux pumps, and biofilm formation. Fosfomycin alone and in combination with amikacin and ciprofloxacin is effective against MDR *E*.* coli*. We conducted this study to evaluate the in vitro activity of fosfomycin, alone and in combination with amikacin and ciprofloxacin, against clinical MDR *E. coli* isolates, including ESBL and biofilm producers. We assessed the in vitro effects by calculating the minimum inhibitory concentration (MIC). The biofilm grades were evaluated using a spectrophotometer. We also assessed the drug interactions of fosfomycin with amikacin and ciprofloxacin using the fractional inhibitory concentration index (FICI) and Epsilometer test (E-test) cross methods.

Methods: This study was conducted from April 2023 to March 2025 at Kalinga Institute of Medical Sciences (KIMS), Bhubaneswar, India. We included MDR *E. coli *isolates collected from clinical specimens (i.e., urine, blood, cerebrospinal fluid, respiratory samples, pus, and tissues) of adult patients of both sexes. The bacteria in the specimens were cultivated using enriched and selective media, such as MacConkey agar and 5% sheep blood agar. Antibiotic susceptibility pattern of *E*.* coli* evaluated by VITEK AST card N-235 and N-405 (BioMérieux, Marcy-l'Étoile, France) for urinary and non-urinary isolates, respectively. The MDR strains were noted. The biofilm grades were assessed using a spectrophotometer by measuring optical density (OD). The higher OD values were suggestive of stronger biofilms by the MDR *E. coli *isolates. The drug interactions of fosfomycin with amikacin and ciprofloxacin were assessed through FICI and E-test cross methods. Lower FICI values indicated additive and synergistic interactions. We analyzed the data through R software (version 4.5.2) (R Foundation for Statistical Computing, Vienna, Austria).

Results: We analyzed 422 MDR *E. coli *isolates during the study period. The majority of the samples were urine samples (306, 72.5%). Most of the MDR *E. coli* isolates were ESBL producers (364, 86.3%). Biofilm production was seen in only 131 (31.0%) isolates. The median OD value of all 422 isolates was 0.073 (0.066-0.129). The majority of isolates were either biofilm non-producers or weak biofilm producers. The MIC90 values of fosfomycin, amikacin, and ciprofloxacin were 32, 8, and 0.5 micrograms/ml, respectively. The FICI values of the combinations of fosfomycin with amikacin and ciprofloxacin were 0.62 and 0.86 microgram/ml, respectively. These values supported the synergistic and additive interactions among the drugs. Drug combinations were more effective than monotherapy across all categories of biofilm producers.

Conclusion: The MDR *E. coli *isolates were more common in urine specimens. Most of them were ESBL producers. Around one-third of isolates were biofilm producers. The drug combinations were more effective than monotherapy with the three antimicrobials. We recommend prospective studies with larger sample sizes to confirm the clinical relevance of our findings.

## Introduction

*Escherichia coli* is a facultative anaerobic, gram-negative (GN) bacillus of the Enterobacteriaceae family. It causes urinary tract infections (UTIs), intestinal and bloodstream infections, and surgical site infections [1-3]. Non-susceptibility to at least one antibiotic in three or more classes, as determined by in vitro susceptibility testing, is referred to as multidrug resistance [4]. β-lactamases, enzymes that hydrolyze β-lactam antibiotics and render them ineffective, are typically responsible for mediating β-lactam resistance [5]. Infections caused by multidrug-resistant (MDR) *E. coli* are responsible for millions of deaths worldwide [6,7]. MDR *E. coli* possess multiple resistance mechanisms (e.g., extended-spectrum beta-lactamase (ESBL) production, efflux pumps, and biofilm formation). Surveillance of ESBL-producing *E. coli* is crucial for directing empirical treatment and tracking resistance patterns [6-9].

Biofilm formation complicates the management of infectious conditions. Because the biofilms protect bacteria from antimicrobials and host defenses, thereby promoting persistence and recurrent infections. *E. coli* biofilms are particularly relevant in catheter-associated UTIs and healthcare settings, and their association with MDR phenotypes contributes to therapeutic challenges [8,10,11]. Approximately 67% of uropathogenic isolates of MDR *E. coli* strains produce biofilms. Biofilms are highly resistant to antibiotics owing to limited drug penetration and reduced metabolism [10-12]. The biofilm matrix, composed of extracellular DNA, proteins, and polysaccharides, protects bacteria from antimicrobials and host defenses, thereby promoting persistence and recurrent infections [12,13].

This highlights the need to explore the drugs with anti-biofilm effects. Antibacterial agents, such as fosfomycin, have a distinct mechanism of inhibiting MurA enzyme-mediated peptidoglycan synthesis, making them effective against MDR and ESBL-producing *E. coli* strains [14]. It has activity against biofilm-associated bacteria and can be used in combination with bactericidal antibiotics such as ciprofloxacin and amikacin [14,15]. Fosfomycin combined with amikacin or ciprofloxacin targets distinct bacterial pathways, thereby improving bacterial killing and biofilm penetration [14-16]. Their different targets increase the potential for synergistic interaction and reduce the likelihood of cross-resistance [15,16]. Evaluating drug interactions of these drugs using the fractional inhibitory concentration index (FICI) [17] and Epsilometer test (E-test) cross methods [18] helps identify clinically useful combinations for MDR *E. coli* isolates.

An in vitro study was conducted as a preliminary step to evaluate the antimicrobial efficacy and synergistic potential of fosfomycin combinations before clinical application. Such studies are essential for screening promising antibiotic combinations, determining MICs, and assessing synergy indices under controlled conditions. Given the rising prevalence of MDR *E. coli* in India and limited treatment options, this study was undertaken to evaluate the in vitro activity of fosfomycin, alone and in combination with amikacin and ciprofloxacin, against clinical MDR *E. coli* isolates, including ESBL producers and biofilm producers. We assessed the in vitro effects by calculating the minimum inhibitory concentration (MIC). The grade of biofilm production was evaluated by its optical density (OD) values. We gauged the drug interactions of fosfomycin with amikacin and ciprofloxacin through alluvial plots.

## Materials and methods

Study design

This in vitro study was conducted in the Department of Microbiology at Kalinga Institute of Medical Sciences (KIMS), Bhubaneswar, from April 2023 to March 2025. Before conducting the study, we obtained ethical permission from the Institutional Ethics Committee (KIIT/KIMS/IEC/1199/2023 dated 27.02.2023).

Study criteria

Clinically significant (i.e., with confirmed infections), non-duplicate MDR *E. coli* isolates collected from various clinical specimens (urine, blood, pus, respiratory samples, cerebrospinal fluid, and tissues) of adult patients (admitted to the hospital during the study period) of both sexes were included. A single specimen was analyzed for each participant. Repeat isolates, colonizers, environmental samples, and isolates with incomplete clinical data were excluded.

Study procedure

The study was carried out on non-repeat clinically relevant isolates of MDR *E. coli* during the study period. Demographic details (age and sex) and specimen type were noted. The clinical samples were collected in sterile containers following aseptic measures and sent to the laboratory without delay. Urine samples were inoculated into Cysteine Lactose Electrolyte Deficient (CLED) media. The plates were then incubated at 37°C overnight. Blood samples were collected in BacT/Alert (BioMérieux, Marcy-l'Étoile, France) broth bottles. The bottles were incubated in a BacT/Alert 3D incubator (BioMérieux, Marcy-l'Étoile, France) for five days or until the bottles were flagged as positive. Then, plating was done on blood agar and MacConkey agar. Respiratory (sputum, endotracheal aspirate, and bronchoalveolar lavage fluid), pus, and tissue samples were also inoculated onto MacConkey agar and blood agar and incubated in a CO_2_ incubator for 24 to 48 hours.

**Figure 1:**
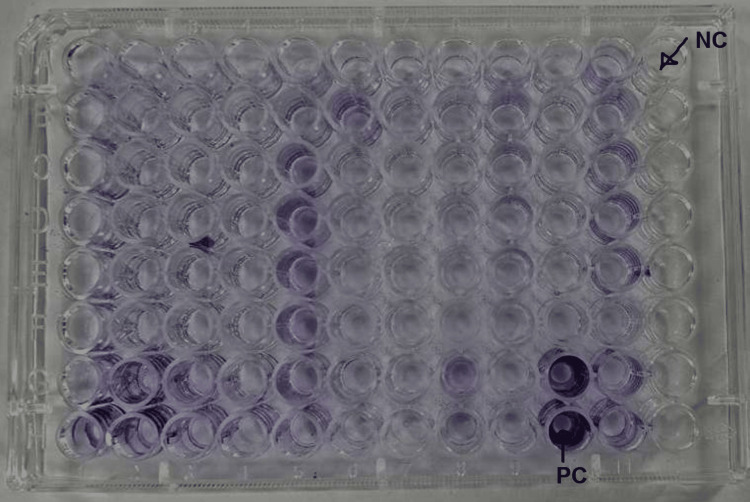
Biofilm formation in 96-well microplates. NC: negative control, PC: positive control.

The isolated colonies of *E. coli* were identified using the VITEK 2 automated system (BioMérieux, Marcy-l'Étoile, France) with the GN card. Antibiotic susceptibility pattern of *E. coli* evaluated by VITEK AST card N-405 for non-urinary isolates and N-235 for urinary isolates. MDR strain was defined as non-susceptibility to at least one agent in three or more antimicrobial categories. MDR *E. coli* were stored in 50% glycerol broth for testing ESBL production, biofilm formation, fosfomycin sensitivity, and the synergistic effects of fosfomycin combined with amikacin and ciprofloxacin. The tissue culture plate method for biofilm formation (Figure 1) was performed using 96-well microplates [19]. For quality control, *E. coli* (ATCC 25922) and *K. pneumoniae* (ATCC 700603) were used as negative and positive controls [20]. The MDR isolates were grown overnight in tryptic soy broth (TSB) with 1% glucose at 37°C. 200 μL of bacterial suspension was inoculated into sterile 96-well flat-bottom polystyrene microtiter plates. After 24 hours of incubation at 37°C, the wells were washed with phosphate-buffered saline (PBS, pH 7.2). Then they were fixed with methanol for 15 min and stained with 0.1% crystal violet for 15 min. The excess stain was washed off, wells were dried, and dye was solubilized with 33% glacial acetic acid. The optical density (OD₄₉₀) of stained adherent bacteria was measured using a microplate reader at 490 nm [21]. The classification of biofilm formation capacity is shown in Table 1. The optical density cut-off value (ODc) is defined as three times the standard deviation (SD) above the mean OD value of the negative control.

**Table 1:** Interpretation of biofilm formation capacity. ODs: mean optical density of the sample. ODc: mean OD of the negative control plus three times the standard deviation (SD).

Criteria	Biofilm formation capacity
ODs ≤ ODc	Not a biofilm producer
ODc ≤ ODs ≤ 2 × ODc	Weak biofilm producer
2 × ODc ≤ ODs ≤ 4 × ODc	Moderate biofilm producer
4 × ODc < ODs	Strong biofilm producer

**Figure 2:**
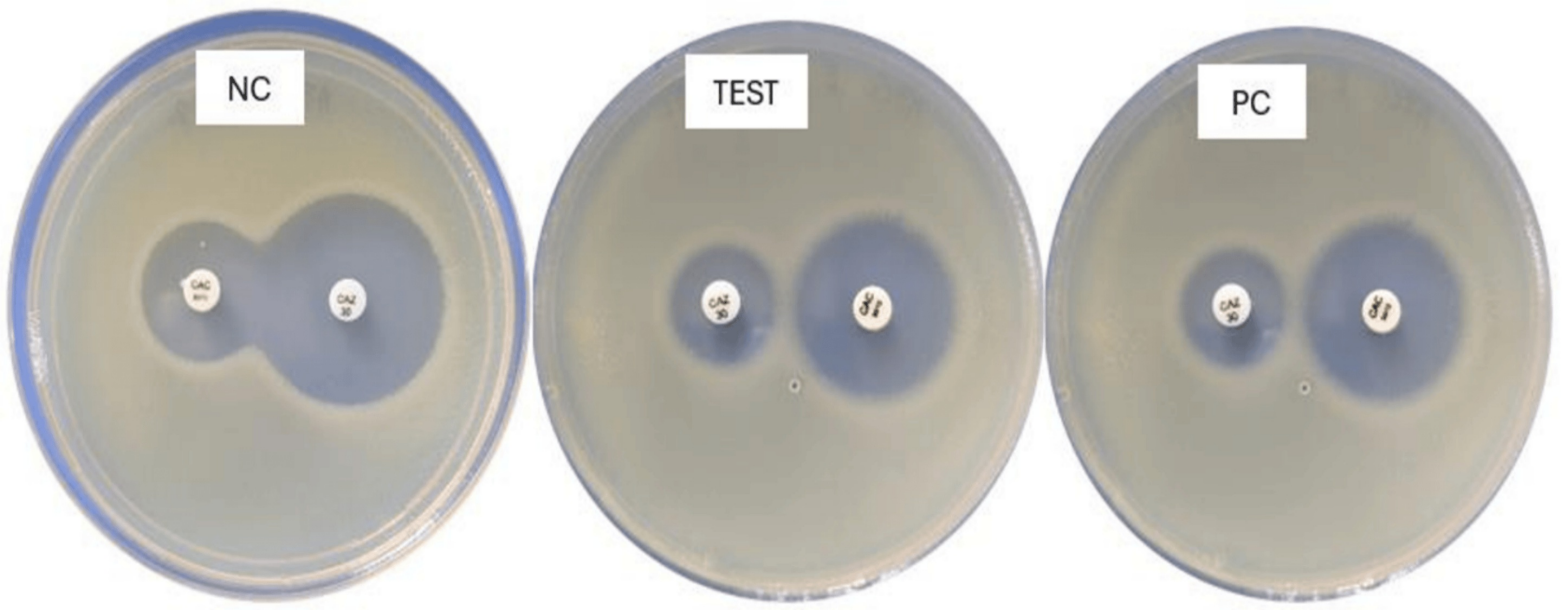
Detection of ESBL production by the combined disk method. ESBL: extended-spectrum beta-lactamase, NC: negative control, PC: positive control.

The detection of ESBL producers (Figure 2) was screened by reduced susceptibility to ceftazidime 30 μg and cefotaxime 30 μg. The production of ESBL was confirmed by the combined disk test (CDT). The CDT employs ceftazidime 30 μg versus ceftazidime 30 μg + clavulanate 10 μg. An increase of ≥5 mm in zone diameter with clavulanate was considered ESBL positive. For quality control, *E. coli* ATCC 25922 and *K. pneumoniae* ATCC 700603 were used as negative and positive controls, respectively.

**Figure 3:**
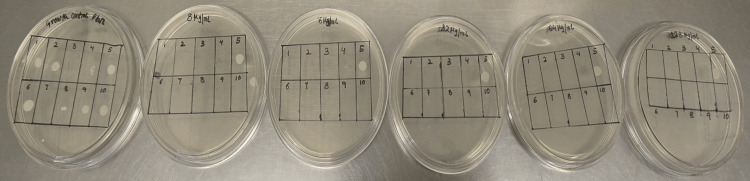
MIC determination by agar dilution method. MIC: minimum inhibitory concentration.

The in vitro activity of fosfomycin against MDR *E. coli* was tested using the agar dilution method (Figure 3) as per Clinical and Laboratory Standards Institute (CLSI) guidelines [22]. Different concentrations of fosfomycin (i.e., 8, 16, 32, 64, and 128 µg/ml) were prepared. The bacterial isolates were spot-inoculated onto these drug-containing plates, and a separate growth-control plate was incubated at 37°C for 24 hours. After incubation, growth was assessed on the control plate to confirm the bacteria's viability. The lowest concentration of fosfomycin at which no visible bacterial growth occurred was recorded as the MIC for that isolate. The MIC90 value represents the MIC that inhibits the growth of 90% of the organisms.

The effect of fosfomycin on biofilm disruption by MDR *E. coli* was tested in 96-well plates. After biofilm formation, the wells were washed to remove free-floating bacteria, then treated with fosfomycin at concentrations ranging from 0.25 µg/mL to 32 µg/mL. Following 24 hours of incubation at 37°C, the wells were washed, stained with crystal violet, and the optical density (OD₄₉₀) was measured to assess biofilm mass. Each test was done in triplicate for accuracy [21].

**Figure 4:**
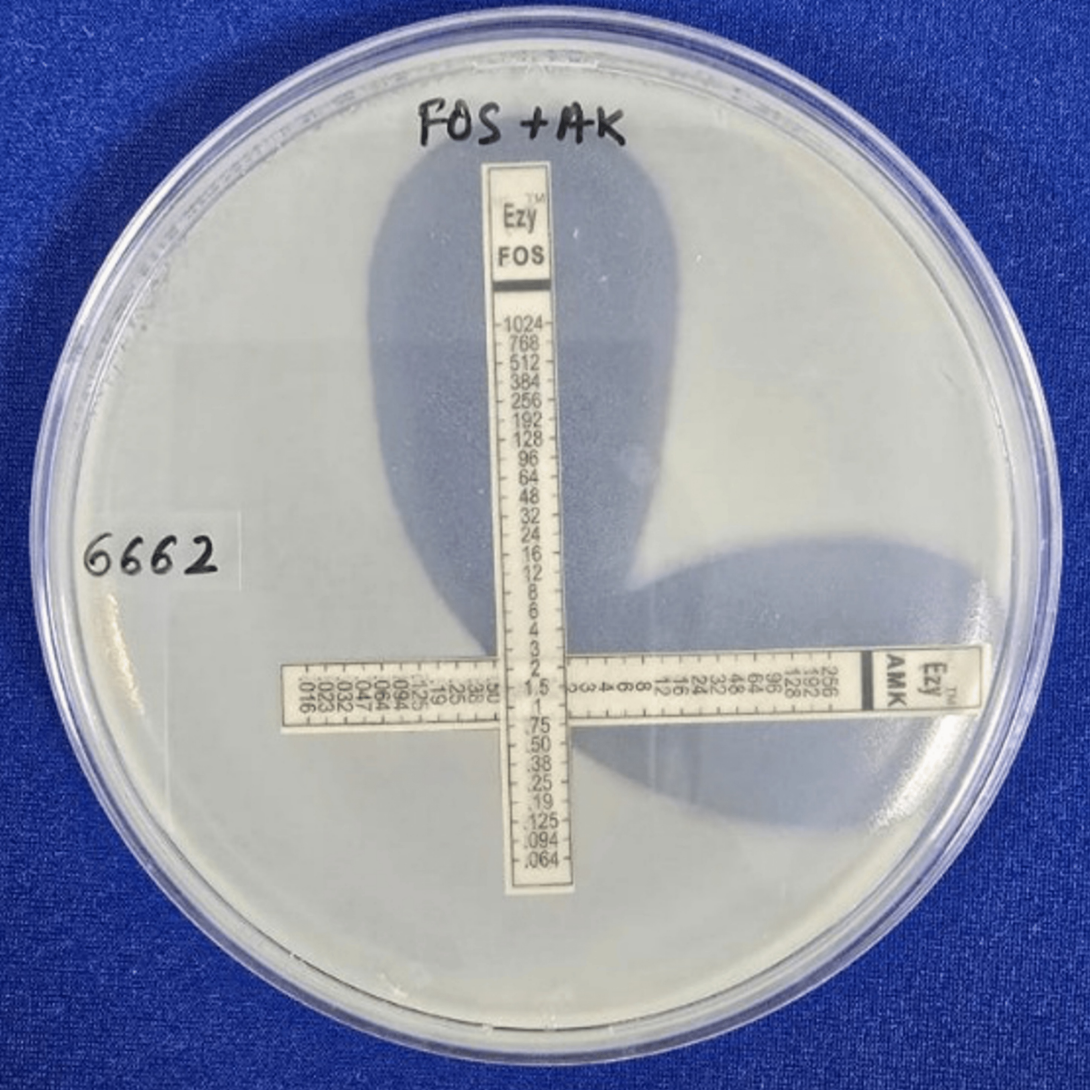
Synergy testing of fosfomycin with amikacin.

**Figure 5:**
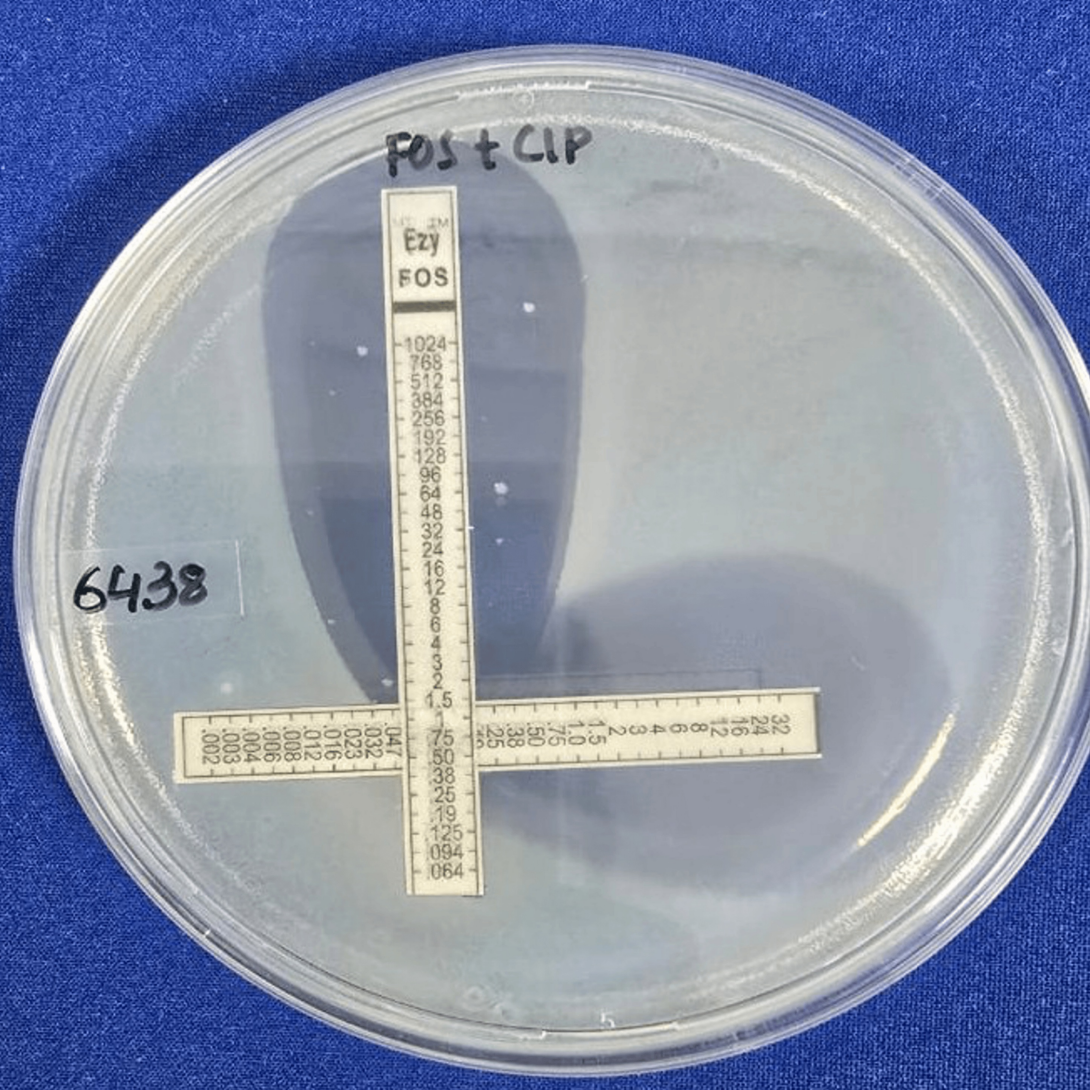
Synergy testing of fosfomycin with ciprofloxacin.

The MIC values of ciprofloxacin and amikacin for synergy testing were confirmed by the Epsilometer test (E-test) strip [18]. Inoculum of 0.5 McFarland was lawn cultured over a Mueller-Hinton agar (MHA) plate, and E-test strips of ciprofloxacin and amikacin were placed individually over them. The plates were incubated at 35-37°C in an incubator for 18 hours. The point at which the zone of inhibition meets the strip was determined to be the MIC. The in vitro activity of combination antibiotics was tested by placing E-test strips of the two antimicrobials on agar at a 90° angle, with the intersection at the respective MICs for the organism. Figures 4, 5 demonstrate the drug interactions of fosfomycin with amikacin and ciprofloxacin, respectively. The MHA agar plates were incubated at 35-37°C in an incubator for 18 hours, and the MICs of each antimicrobial combination were determined. The effects of the antimicrobial combinations were defined according to the FICI [17]. The formula for FICI is as follows:

FICI = (MIC drug A plus drug B/MIC drug A) + (MIC drug A plus drug B/MIC drug B).

Table 2 illustrates the interpretation of FICI values. Figures 4, 5 show the synergy testing of fosfomycin with amikacin and ciprofloxacin, respectively.

**Table 2:** Interpretation of FICI values. FICI: fractional inhibitory concentration index.

FICI value	Interpretation
≤0.5	Synergy
>0.5 to ≥1	Additive
>1 to ≤4	No interaction (indifference)
>4	Antagonism

Statistical analysis

A non-probability consecutive sampling was used to conduct this in vitro study. The Shapiro-Wilk test was used to verify that the data were normally distributed. The median and interquartile range (IQR) were used to describe the continuous data. For the categorical data, the frequency and proportion were shown. We used an upset plot to illustrate the distribution of the study population. The OD values of biofilms were depicted through a combination of half-eye, beeswarm, and jitter plots. The alluvial plots illustrated drug interactions between fosfomycin and amikacin or ciprofloxacin across various biofilm types and ESBL producers. R software version 4.5.2 (R Foundation for Statistical Computing, Vienna, Austria) was used to compute the data [23]. Statistical significance was defined as a p-value of 0.05 or less.

## Results

In this study, we analyzed 422 clinical isolates of MDR *E. coli*. Table 3 shows the demographic and clinical traits of the study participants. 221 (52.4%) of 422 participants were males. The median age of the study population was 53.5 (38.5-71.0) years. The 306 (72.5%) urine samples accounted for the majority. There were 131 (31.0%) biofilm producers and 364 (86.3%) ESBL producers.

**Table 3:** Demographic and clinical traits of the study population. The median (IQR) was used to depict the continuous variables. n (%) was used to display the category values. IQR: interquartile range, ESBL: extended-spectrum beta-lactamase.

Parameters	Value
Total participants	422
Age (years)	53.5 (38.5-71.0)
Elderly	147 (34.8%)
Male	221 (52.4%)
Urine samples	306 (72.5%)
Biofilm producers	131 (31.0%)
ESBL producers	364 (86.3%)
Duration of hospitalization (days)	12.5 (6.5-18.5)

**Figure 6:**
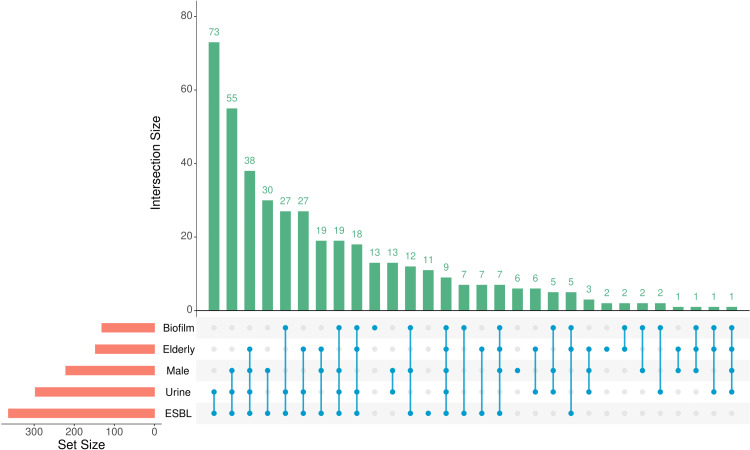
Upset plot showing the distribution of the study population. The distribution of the study population is illustrated through an upset plot. The horizontal bar plot (red) shows the number of participants across the five sets (i.e., male and elderly participants, urine samples, ESBL-producing pathogens, and biofilm producers). The matrix (blue color) shows various intersections through the highlighted dots and connecting lines. The vertical bar plot (green) shows the number of participants in each intersection. Both bar plots are arranged in decreasing order. ESBL: extended-spectrum beta-lactamase.

Figure 6 illustrates the distribution of the study population through an upset plot. The upset plot consists of two sets of bar plots (one horizontal and one vertical) and a matrix that demonstrates the intersections of the variables shown in the horizontal bar plot. The following data were shown in the horizontal bar plot: males, elderly participants, urine samples, ESBL-producing pathogens, and biofilm producers. Both bar plots were arranged in decreasing order. Urine samples from 73 (17.3%) young female and 55 (13.0%) young male subjects yielded ESBL-producing, biofilm-nonproducing MDR *E. coli* isolates.

**Figure 7:**
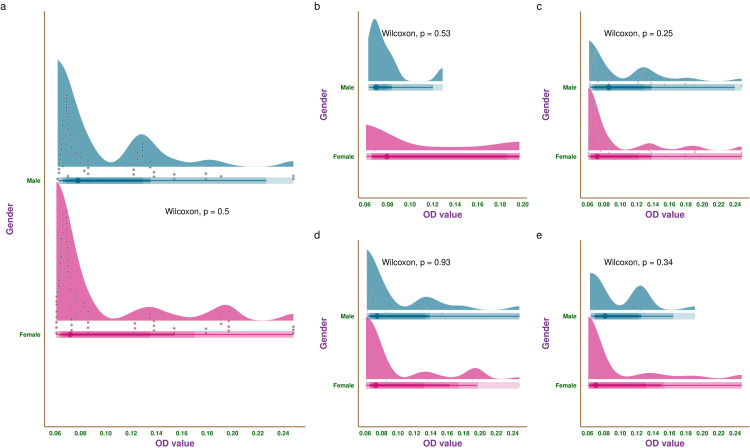
OD values of the biofilms produced by E. coli isolates. The half-eye, interval, and beeswarm plots illustrate the OD values of biofilms produced by the *E. coli* isolates. Figures (a)-(e) represent the entire study population, participants without ESBL-producing *E. coli*, participants with ESBL-producing *E. coli*, adult participants, and elderly participants, respectively. OD: optical density, ESBL: extended-spectrum beta-lactamase.

Figure 7 illustrates the OD values of biofilms produced by the *E. coli* isolates through half-eye, interval, and beeswarm plots. Figure 7a shows the median OD values of the entire study population. The median OD value was 0.073 (0.066-0.129) (male: 0.078 (0.069-0.129), female: 0.072 (0.066-0.135), p = 0.50). Figure 7b shows the median OD values of the study participants without ESBL-producing *E. coli* (n = 58). The median OD value of this subgroup was 0.076 (0.066-0.126) (male: 0.070 (0.069-0.080), female: 0.080 (0.066-0.186), p = 0.53). Figure 7c shows the median OD values of the study participants with ESBL-producing *E. coli* (n = 364). The median OD value of this subgroup was 0.073 (0.066-0.129) (male: 0.086 (0.069-0.129), female: 0.072 (0.065-0.123), p = 0.25). Figure 7d shows the median OD values of adult participants (n = 275). The median OD value of this subgroup was 0.073 (0.066-0.135) (male: 0.075 (0.067-0.133), female: 0.073 (0.067-0.132), p = 0.93). Figure 7e shows the median OD values of elderly participants (n = 147). The median OD value of this subgroup was 0.070 (0.066-0.123) (male: 0.082 (0.069-0.122), female: 0.070 (0.063-0.132), p = 0.34). The ODc value in our study was 0.04. Based on the criteria for OD values shown in Table 1, the majority of isolates were either biofilm non-producers or weak biofilm producers.

Table 4 shows the MIC values of the three drugs (i.e., fosfomycin, amikacin, and ciprofloxacin) alone and the FICI values of the two combinations (i.e., fosfomycin plus amikacin and fosfomycin plus ciprofloxacin). The lower FICI values for combinations of two drugs (amikacin and ciprofloxacin) with fosfomycin indicate synergistic and additive interactions among the drugs.

**Table 4:** MIC90 and FICI values. The MIC90 values were expressed in micrograms per milliliter. MIC: minimum inhibitory concentration, FICI: fractional inhibitory concentration index, ESBL: extended-spectrum beta-lactamase.

Variables	MIC90 fosfomycin	MIC90 amikacin	MIC90 ciprofloxacin	FICI for fosfomycin plus amikacin	FICI for fosfomycin plus ciprofloxacin
Total (n = 422)	32	8	0.5	0.62	0.86
Male (n = 221)	32	8	0.5	0.57	0.78
Female (n = 201)	32	8	0.5	0.68	0.95
ESBL positive (n = 364)	32	8	0.5	0.61	0.84
ESBL negative (n = 58)	32	8	0.5	0.68	0.99
Biofilm producers (n = 131)	32	8	0.5	0.56	0.79
Biofilm non-producers (n = 291)	32	8	0.5	0.65	0.89
Urine samples (n = 306)	32	8	0.5	0.57	0.83
Non-urine samples (n = 116)	64	16	1	0.75	0.94

**Figure 8:**
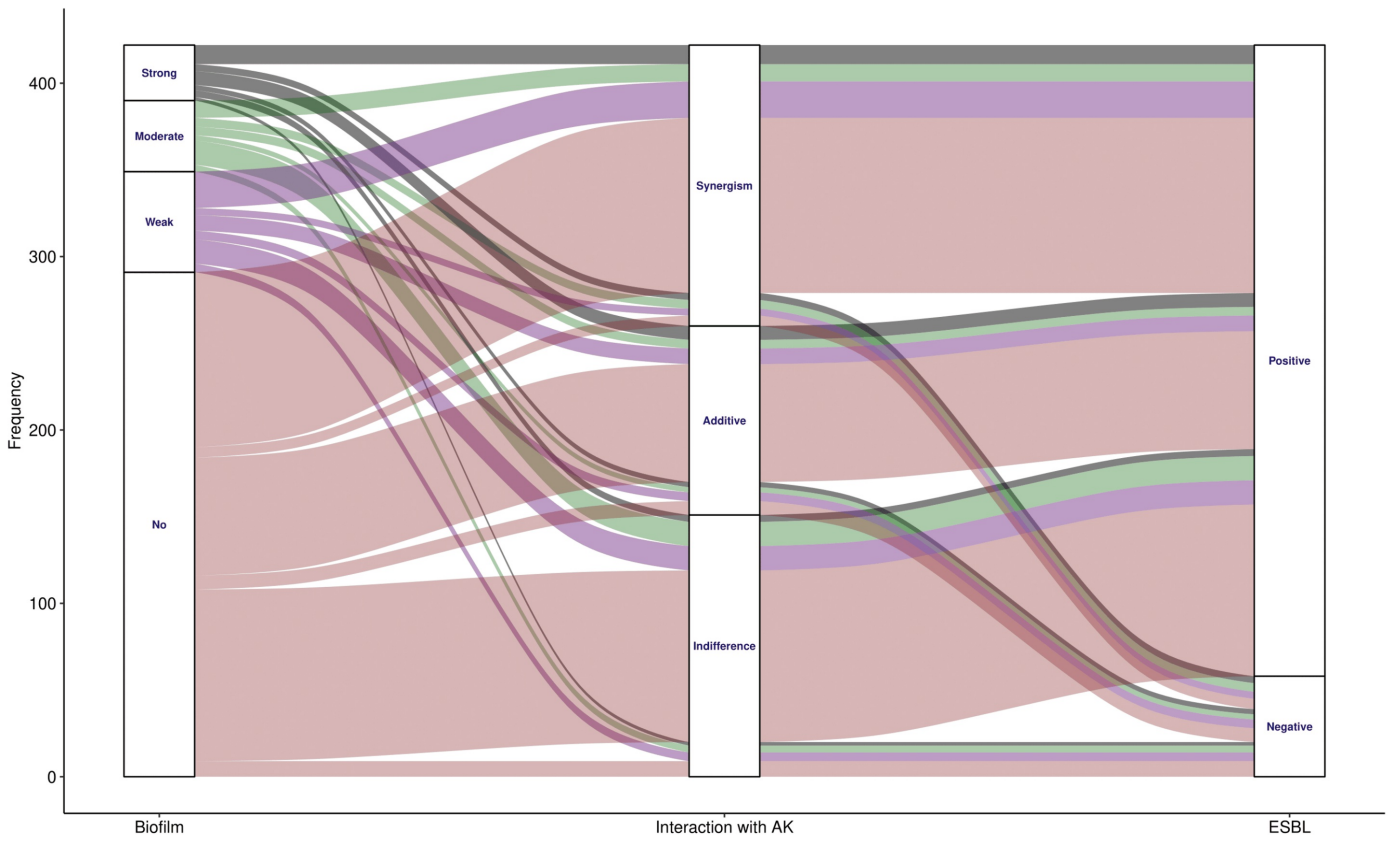
Associations of biofilm and ESBL production with interaction of fosfomycin and amikacin. The alluvial plot illustrates the association between biofilm and ESBL production in MDR *E. coli*, as well as the interactions between fosfomycin and amikacin. The number of participants is shown on the y-axis. The biofilm types (i.e., strong, moderate, weak, and no biofilm) are portrayed in different colors. The widths of the bands denote the proportions of participants with the relevant parameters. ESBL: extended-spectrum beta-lactamase, MDR: multidrug-resistant, AK: amikacin.

**Figure 9:**
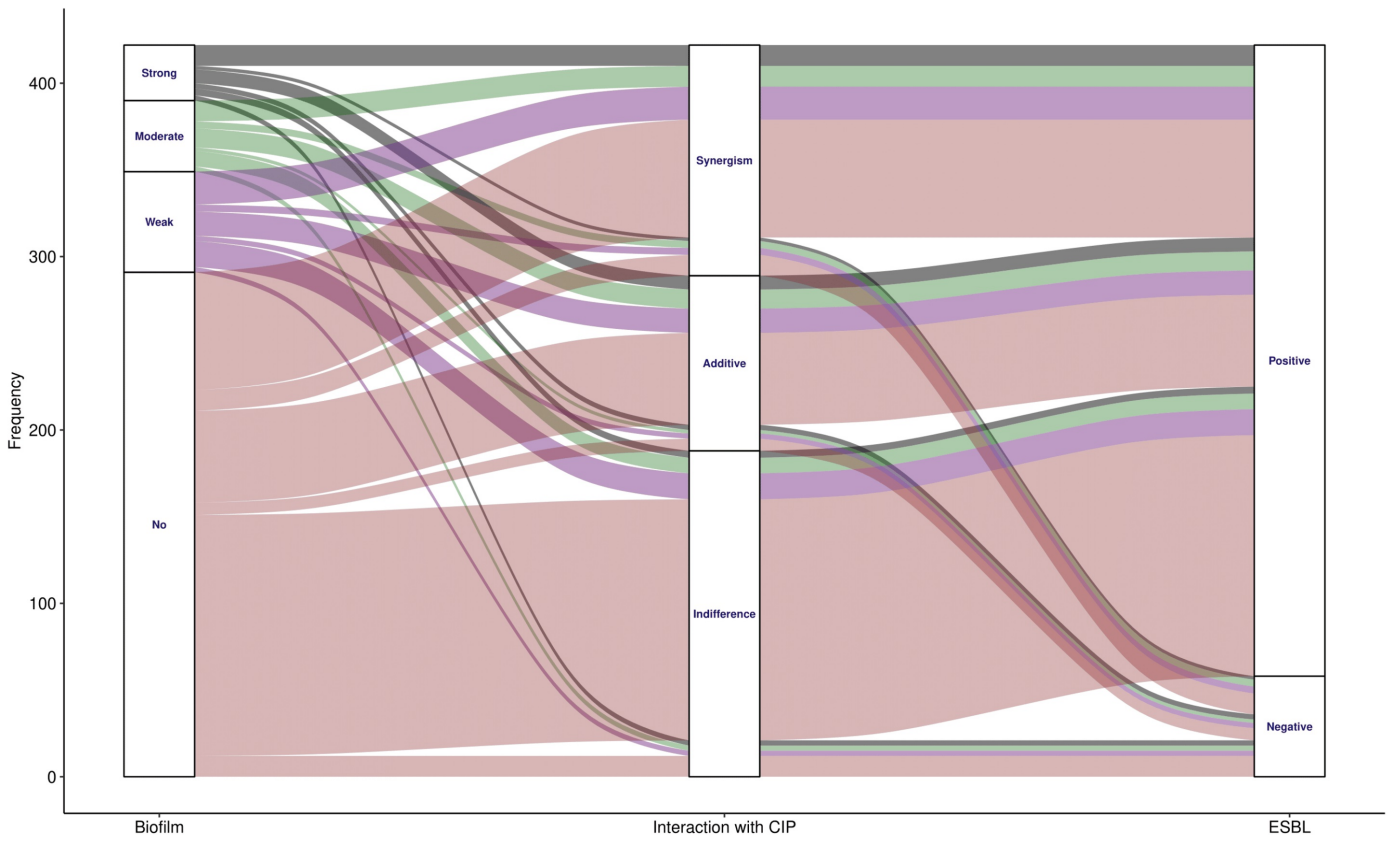
Associations of biofilm and ESBL production with interaction of fosfomycin and ciprofloxacin. The alluvial plot illustrates the association between biofilm and ESBL production in MDR *E. coli*, as well as the interactions between fosfomycin and ciprofloxacin. The number of participants is shown on the y-axis. The biofilm types (i.e., strong, moderate, weak, and no biofilm) are portrayed in different colors. The widths of the bands denote the proportions of participants with the relevant parameters. ESBL: extended-spectrum beta-lactamase, MDR: multidrug-resistant, CIP: ciprofloxacin.

The alluvial plots in Figures 8, 9 illustrate the association among fosfomycin-drug interactions (with amikacin and ciprofloxacin), biofilm grades, and the presence of ESBL-producing *E. coli*. There were 131 (31.0%) biofilm-producing and 364 (86.3%) ESBL-producing MDR *E. coli*. Figure 8 shows 158 (37.4%) cases of synergism and 113 (26.8%) cases of additive interactions between fosfomycin and amikacin. The remaining 151 (35.8%) interactions were insignificant. The incidence of synergism was higher than that of additive interactions across all three biofilm categories (i.e., strong, moderate, and weak). Figure 9 shows 133 (31.5%) cases of synergism and 96 (22.7%) cases of additive interactions between fosfomycin and ciprofloxacin. The remaining 193 (45.8%) interactions were insignificant. The incidence of synergism was higher than that of additive interactions across all three biofilm categories (i.e., strong, moderate, and weak). Combinations of fosfomycin with amikacin and ciprofloxacin were more effective (as evidenced by synergistic interactions) than monotherapy for all categories of biofilm producers.

## Discussion

In this in vitro study, we analyzed 422 MDR *E. coli* isolates (one isolate per participant). The majority of the samples were urine samples (306, 72.5%). Most of the MDR *E. coli* isolates were ESBL producers (364, 86.3%). Biofilm production was seen in only 131 (31.0%) isolates. The FICI values were lower for the following MDR *E. coli* isolates than for their counterparts: ESBL producers, biofilm producers, and isolates obtained from urine specimens or male participants. Lower FICI values suggested synergistic and additive interactions among the drugs. Our observations concurred with the findings of two recent studies [21,24,25].

Among the 422 samples analyzed, the urine samples from 73 (17.3%) young female and 55 (13.0%) young male subjects had the highest numbers of MDR *E. coli* isolates. An equilibrium between the urethra and bacteria can be maintained by the urethral mucosa and epithelial cells' ability to fight off harmful bacterial invasion. Highly pathogenic bacteria, such as uropathogenic *E. coli* (UPEC), disrupt this equilibrium and weaken the defense system. These reactions are frequently influenced by specific UPEC components, including polysaccharide capsules, flagella, fimbriae, outer-membrane proteins (OMPs), lipopolysaccharides (LPSs), and iron-acquisition receptors [1,8,9].

MDR *E. coli* exerts antimicrobial resistance through multiple mechanisms (e.g., ESBL production, biofilm formation, and efflux pumps). Biofilms not only shield pathogens from phagocytosis, antimicrobial peptides, and antibody opsonization but also prevent bacteria from being ejected by epithelial cells via ciliary action [6,7,26]. Numerous efflux pumps, which are responsible for eliminating a variety of chemicals from inside the cell, are frequently encoded in bacterial genomes. Efflux pumps can significantly affect a bacterial species' innate drug sensitivity and the emergence of clinically meaningful antibiotic resistance when overexpressed [7-9,26,27].

The FICI values and alluvial plots revealed that drug combinations were more effective than monotherapy with the three antimicrobials. Studies in animals have shown that fosfomycin prevents aminoglycoside-induced histamine release from mast cells, thereby providing protection against aminoglycoside-related nephrotoxicity [14,15,28]. Because of amikacin's concentration-dependent bactericidal activity, this combination will enable a higher, once-daily amikacin dosage if required [15,28].

Strengths and limitations

The calculations of OD value for assessing biofilm strength and FICI values for interpreting the drug interactions were the strengths of this study. Another plus was the graphical illustration of drug interactions through the alluvial plots. Our study had some limitations as well. First, clinical correlation could not be performed owing to the in vitro study design. Secondly, we analyzed neither non-MDR *E. coli* isolates nor any other MDR pathogens.

## Conclusions

Urine specimens had more MDR *E. coli* isolates. They were mostly ESBL producers. Biofilms were produced by about one-third of the isolates. The medication combinations outperformed the three antimicrobials when used alone, as evidenced by the FICI values. Combinations of fosfomycin with amikacin and ciprofloxacin were more effective (as evidenced by synergistic interactions) than monotherapy for all categories of biofilm producers. To clinically correlate the results of our study, we advise conducting prospective studies with larger sample sizes.
